# Supplementary material for: Mental-somatic multimorbidity in trajectories of cognitive function for middle-aged and older adults
Source: PLoS One. 2024 May 14;19(5):e0303599. doi: 10.1371/journal.pone.0303599 (PMC11093294; doi:10.1371/journal.pone.0303599)
Supplement: S1 Appendix — (DOCX) [file pone.0303599.s001.docx]

**S1 Appendix.** Comprehensive Analysis Documentation.

1. **Study population**

**Figure S1**. Sample Size Flow Diagram

**Table S1.** Descriptive table for distribution of the trajectory groups by levels of independent variables and covariates

1. **Group-based trajectory model (GBTM) selection process**

**Table S2.** Fit statistics for the two to six basic group trajectory model

**Figure S2.** Trajectory plots for the two to six basic group trajectory model

1. **GBTM extended to account for attrition**

**Table S3.** The odds ratio of dropout by multimorbidity (MM) category for each group in full group-based trajectory model

**Figure S3.** Estimated dropout probabilities for each group in full group-based trajectory model

**Table S4.** Fit statistics for the group-based trajectory model not accounting for attrition

**Table S5.** Odds of cognitive impairment by multimorbidity category in the group-based trajectory model not accounting for attrition

1. **Sensitivity analysis between multinomial regression model with and without person’s posterior probability of group membership as weights**

**Table S6.** The multinomial logistic regression model with person’s posterior probability of group membership as weights

1. **Predicted probabilities of cognitive impairment at decades of age in the full group-based trajectory model (GBTM)**

**Table S7.** Predicted probabilities of cognitive impairment at decades of age in the full model

1. **Study population**

**Figure S1. Sample Size Flow Diagram**

The current study followed all HRS participants starting from the earliest age of cohort eligibility until dropout/death. Participants over 90 years old were excluded due to the sparsity in the data. Of 36,170 HRS respondents interviewed between 1998 and 2016 who were alive, living in the community and cohort-eligible (i.e., respondents with a positive survey weight), A total of 481 respondents were excluded from the study due to all survey waves being completed after the age of 90. So 35,689 respondents were included who gave their interviews at age 51–90 during eligible survey waves. We excluded 1,257 respondents who used a proxy respondent due to missing assessment of depression during all eligible interviews. We then excluded 1,320 respondents who reported other race (other races include American Indian, Alaskan Native, Asian, Native Hawaiian, and Pacific Islander), or with no information on race and 428 respondents with any missing data on other sociodemographic and health-related covariates. We also excluded 3,609 respondents with inconsistent chronic disease patterns (i.e., “yes” followed by “no” at subsequent waves). Lastly, an additional 9,005 participants with fewer than 3 assessments of cognitive function and/or chronic diseases, as required for adequate modeling of temporal trajectories, were also excluded. As a result, the final analytic sample consisted of 20,070 respondents.

**Table S1. Descriptive table for distribution of the trajectory groups by levels of independent variables and covariates**

|  | **Total** | **Low risk with late-life increase** | **Low initial risk with rapid increase** | **High risk** | **p value^*^** |
| --- | --- | --- | --- | --- | --- |
| **N (%)** | 20070 | 12560 (62.6) | 5155 (25.7) | 2355 (11.7) |  |
| **Baseline age (mean (SD))** | 61.0 (8.7) | 60.3 (8.5) | 62.9 (9.1) | 60.7 (7.9) | <0.01 |
| **Sex, n (%)** |  |  |  |  | <0.01 |
| Male | 8522 | 5222 (61.3) | 2221 (26.1) | 1079 (12.7) |  |
| Female | 11548 | 7338 (63.5) | 2934 (25.4) | 1276 (11.1) |  |
| **Race/ethnicity, n (%)** |  |  |  |  | <0.01 |
| Hispanic | 2320 | 1032 (44.5) | 710 (30.6) | 578 (24.9) |  |
| NH White | 14046 | 10042 (71.5) | 3234 (23.0) | 770 (5.5) |  |
| NH Black | 3704 | 1486 (40.1) | 1211 (32.7) | 1007 (27.2) |  |
| **Education, n (%)** |  |  |  |  | <0.01 |
| < High school | 4166 | 1242 (29.8) | 1533 (36.8) | 1391 (33.4) |  |
| High school graduate | 10566 | 6863 (65.0) | 2864 (27.1) | 839 (7.9) |  |
| College | 5338 | 4455 (83.5) | 758 (14.2) | 125 (2.3) |  |
| **Wealth quartiles, n (%)** |  |  |  |  | <0.01 |
| 1^st^ quartile (lowest) | 5462 | 2472 (45.3) | 1643 (30.1) | 1347 (24.7) |  |
| 2^nd^ quartile | 4922 | 2861 (58.1) | 1515 (30.8) | 546 (11.1) |  |
| 3^rd^ quartile | 4877 | 3416 (70.1) | 1143 (23.4) | 318 (6.5) |  |
| 4^th^ quartile (highest) | 4809 | 3811 (79.2) | 854 (17.8) | 144 (3.0) |  |
| **Smoking, n (%)** |  |  |  |  | <0.01 |
| Never smoker | 8468 | 5511 (65.1) | 2058 (24.3) | 899 (10.6) |  |
| Past smoker | 7797 | 4976 (63.8) | 1993 (25.6) | 828 (10.6) |  |
| Current smoker | 3805 | 2073 (54.5) | 1104 (29.0) | 628 (16.5) |  |
| **BMI categories, n (%)** |  |  |  |  | <0.01 |
| Underweight | 204 | 125 (61.3) | 53 (26.0) | 26 (12.7) |  |
| Normal weight | 5854 | 3770 (64.4) | 1462 (25.0) | 622 (10.6) |  |
| Overweight | 7874 | 4995 (63.4) | 1998 (25.4) | 881 (11.2) |  |
| Obese | 6138 | 3670 (59.8) | 1642 (26.8) | 826 (13.5) |  |
| **No. of chronic conditions, mean (SD)** | 1.5 (1.3) | 1.4 (1.2) | 1.8 (1.4) | 1.9 (1.5) | <0.01 |
| **Multimorbidity, n (%)** |  |  |  |  | <0.01 |
| No multimorbidity | 11134 | 7561 (67.9) | 2471 (22.2) | 1102 (9.9) |  |
| Somatic multimorbidity | 6245 | 3784 (60.6) | 1756 (28.1) | 705 (11.3) |  |
| Stroke multimorbidity | 537 | 291 (54.2) | 178 (33.1) | 68 (12.7) |  |
| Depressive multimorbidity | 1959 | 860 (43.9) | 658 (33.6) | 441 (22.5) |  |
| Stroke and Depressive multimorbidity | 195 | 64 (32.8) | 92 (47.2) | 39 (20) |  |
| **Cognitive Score, mean (SD)** | 16.1 (4.3) | 17.9 (3.1) | 14.3 (3.8) | 9.9 (3.5) | <0.01 |
| **Cognition category, n (%)** |  |  |  |  | <0.01 |
| Normal | 17057 | 12446 (73) | 3995 (23.4) | 616 (3.6) |  |
| Cognitive impairment (CIND or demented) | 3013 | 114 (3.8) | 1160 (38.5) | 1739 (57.7) |  |
| **Attrition, n (%)** |  |  |  |  | <0.01 |
| No attrition | 12198 | 8120 (66.6) | 2752 (22.6) | 1326 (10.9) |  |
| Attrition | 7872 | 4440 (56.4) | 2403 (30.5) | 1029 (13.1) |  |

1. **Group-based trajectory model (GBTM) selection process**

Following established guidance [1], we began by fitting a sequence of basic group-based trajectory models without time-varying multimorbidity combinations in order to 1) determine the optimal number of trajectory groups and 2) select the most appropriate functional form (intercept only, linear, quadratic, or cubic) of each trajectory group. Model selection was an iterative process based on a combination of following criteria [1]: 1) diagnostic assessments including reduction in Bayesian Information Criterion (BIC), average posterior probability of group membership > 80% for all groups, odds of correct classification (OCC) >5.0; 2) size of the smallest group >10% of total sample; and 3) the ability to capture clinically relevant and distinct developmental trajectories of cognitive impairment risk across the entire observed age span.

We compared between group-based trajectory models with 2-6 groups and varying order of the polynomial to determine the preferred number of groups and the order specifying the shape of each trajectory. After assessment, we believed that the trajectory of cognitive impairment was best modeled as a quadratic function of time. The improvement in BIC leveled off after four groups, but the average posterior probabilities in three out of the four identified trajectory groups were lower than 0.80, and one group only made up 8.5% (less than 10%) of the analytic sample. Compared with the four-group model, the three-group model achieved optimal average posterior probability of group membership with values ranging from 0.82 to 0.86 across the three trajectory groups although its BIC was a little lower. After evaluating all fit statistics and the trajectory groups, we believe three-group model is the optimal choice after balancing between statistics and clinical meaningful trajetories, even though the OCC for group 1 is 3.62, smaller than the ideal scenario >5.0. Therefore, we opted for the three-group model. The fit statistics is shown below in **Table S1**. The trajectories for group trajectory model are shown below in **Figure S2**.

**Table S2. Fit statistics for the two to six basic group trajectory model***

|  | **Average Posterior Probability** | **Odds of Correct Classification** | **Group Size (%)** | **BIC** |
| --- | --- | --- | --- | --- |
| **Two-group model** |  |  |  |  |
| Group 1 | 0.965 | 9.2 | 74.9 | -47215.69 |
| Group 2 | 0.900 | 27.3 | 25.1 |  |
|  |  |  |  |  |
| **Three-group model** |  |  |  | -45976.09 |
| Group 1 | 0.864 | 3.62 | 57.5 |  |
| Group 2 | 0.821 | 14.1 | 30.4 |  |
| Group 3 | 0.856 | 44.4 | 12.0 |  |
|  |  |  |  |  |
| **Four-group model** |  |  |  | -45737.66 |
| Group 1 | 0.715 | 13.2 | 32.7 |  |
| Group 2 | 0.646 | 1.4 | 39.4 |  |
| Group 3 | 0.721 | 11.2 | 19.4 |  |
| Group 4 | 0.822 | 51.3 | 8.5 |  |
|  |  |  |  |  |
| **Five-group model** |  |  |  | -45734.7 |
| Group 1 | 0.672 | 39.9 | 19.9 |  |
| Group 2 | 0.663 | 10.0 | 27.5 |  |
| Group 3 | 0.453 | 0.7 | 27.7 |  |
| Group 4 | 0.693 | 11.1 | 17.1 |  |
| Group 5 | 0.810 | 52.2 | 7.8 |  |
|  |  |  |  |  |
| **Six-group model** |  |  |  | -45676.64 |
| Group 1 | 0.484 | 13.8 | 8.7 |  |
| Group 2 | 0.601 | 1.1 | 37.3 |  |
| Group 3 | 0.616 | 12.9 | 26.2 |  |
| Group 4 | 0.521 | 7.5 | 14.4 |  |
| Group 5 | 0.535 | 17.2 | 7.8 |  |
| Group 6 | 0.704 | 35.9 | 5.6 |  |
| *Basic model – not including time-varying multimorbidity, not accounting for attrition. | | | | |

**Figure S2. Trajectory plots for the two to six basic group trajectory model**

| Two-group model | Three-group model |
| --- | --- |
|  |  |
| Four-group model | Five-group model |
|  |  |
| Six-group model |  |
|  |  |
| *X-axis is centered age (age-51); Y-axis is the predicted probability of cognitive impairment. | |

1. **GBTM extended to account for attrition**
   1. **Modeling process**

After determining the optimal number and functional form of identified trajectory groups, we included time-varying indicators for multimorbidity combination groups to examine their relative impact on observed trajectories. To minimize bias from loss to follow-up, we adjusted this model to account for nonrandom participant attrition in group-based trajectory model according to the guidance provided by Nagin [2]. The attrition process is directly modeled and allows for variation across trajectory groups. Thus, the dropout rates after attrition are estimated for each follow up year within each trajectory group.

The dropout model that can be incorporated in the GBTM is a logistic model of dropout probability for each wave (age in our case). Basic dropout models (without any predictors or covariates) and dropout models with different predictors (1st lag response, 2 lag responses, multimorbidity groups) were tested. The dropout models with 1st lag response, 2 lag responses as predictors had unreasonable estimates for trajectory groups and substantially high standard error for coefficients. After comparing and evaluating between the basic dropout model and the dropout model including time-varying multimorbidity groups as predictors for dropout, the best-fit dropout model is the dropout model with time-varying multimorbidity. The estimates are shown below in **eTable 2**. The estimated dropout probability for each trajectory group is shown below in **Figure S3**.

**Table S3. The odds ratio of dropout by multimorbidity (MM) category for each group in full group-based trajectory model**

| **Group** |  | **Odds Ratio** | **95% CI** | **p value** |
| --- | --- | --- | --- | --- |
| Low risk with late-life increase | Somatic MM | Reference |  |  |
|  | No MM | 0.60 | (0.52, 0.70) | <0.01 |
|  | Stroke MM | 1.38 | (1.13, 1.69) | <0.01 |
|  | Depressive MM | 1.33 | (1.10, 1.59) | <0.01 |
|  | Stroke & Depressive MM | 3.08 | (2.23, 4.27) | <0.01 |
|  |  |  |  |  |
| Low initial risk with rapid increase | Somatic MM | Reference |  |  |
|  | No MM | 0.57 | (0.47, 0.69) | <0.01 |
|  | Stroke MM | 1.60 | (1.25, 2.05) | <0.01 |
|  | Depressive MM | 1.05 | (0.84, 1.32) | 0.66 |
|  | Stroke & Depressive MM | 1.56 | (1.04, 2.35) | <0.05 |
|  |  |  |  |  |
| High risk | Somatic MM | Reference |  |  |
|  | No MM | 0.62 | (0.48, 0.79) | <0.01 |
|  | Stroke MM | 1.22 | (0.81, 1.85) | 0.35 |
|  | Depressive MM | 0.94 | (0.72, 1.22) | 0.64 |
|  | Stroke & Depressive MM | 1.46 | (0.87, 2.44) | 0.15 |

*MM: multimorbidity

**Figure S3. Estimated dropout probabilities for each group in full group-based trajectory model**

Group 1: Low risk with late-life increase; Group 2: low initial risk with rapid increase; Group 3: high risk

- 1. **Sensitivity analysis**

We also conducted sensitivity analyses between model without and model with accounting for attrition. We didn’t find any significant differences between them. The fit statistics (**Table S3**), and estimates for multimorbidity groups (**Table S4**) in the model without accounting for attrition are shown below.

**Table S4. Fit statistics for the group-based trajectory model not accounting for attrition**

|  | **Average Posterior Probability** | **Odds of Correct Classification** | **Group Size (%)** | **BIC** |
| --- | --- | --- | --- | --- |
| **Three-group model** |  |  |  | -45577.21 |
| Low risk with late-life increase | 0.837 | 3.08 | 55.3 |  |
| low initial risk with rapid increase | 0.803 | 11.8 | 32.4 |  |
| high risk | 0.860 | 45.8 | 12.4 |  |

**Table S5. Odds of cognitive impairment by multimorbidity category in the group-based trajectory model not accounting for attrition**

|  | **Low risk with late-life increase** | **low initial risk with rapid increase** | **high risk** |
| --- | --- | --- | --- |
|  | **OR (95% CI)** | **OR (95% CI)** | **OR (95% CI)** |
| Multimorbidity (MM) |  |  |  |
| Somatic MM | Reference | Reference | Reference |
| No MM | 0.77(0.65,0.90) ^**^ | 0.68(0.63,0.74) ^**^ | 0.74(0.65,0.85) ^**^ |
| Stroke MM | 2.05(1.74,2.41) ^**^ | 2.71(2.35,3.14) ^**^ | 3.21(2.16,4.77) ^**^ |
| Depressive MM | 2.00(1.68,2.38) ^**^ | 1.90(1.72,2.11) ^**^ | 1.70(1.45,2.00) ^**^ |
| Stroke & Depressive MM | 3.81(2.82,5.15) ^**^ | 4.35(3.53,5.34) ^**^ | 3.42(2.07,5.65) ^**^ |
| ^**^p<0.01 | | | |

1. **Sensitivity analysis between multinomial regression model with and without person’s posterior probability of group membership as weights**

We didn’t find any significant differences between model with and without weights. The model with person’s posterior probability of group membership as weights is shown below in **Table S5**.

**Table S6. The multinomial logistic regression model with person’s posterior probability of group membership as weights**

|  | **low initial risk with rapid increase** | **high risk** |
| --- | --- | --- |
| **Characteristics** | **OR (95% CI)** | **OR (95% CI)** |
| Race/ethnicity |  |  |
| Non-Hispanic White | Reference | Reference |
| Non-Hispanic Black | 2.50(2.27, 2.75) ^**^ | 6.59(5.78, 7.51) ^**^ |
| Hispanic | 1.66(1.47, 1.86) ^**^ | 2.93(2.51, 3.42) ^**^ |
| Sex |  |  |
| Male | Reference | Reference |
| Female | 0.81(0.75, 0.87) ^**^ | 0.61(0.55, 0.68) ^**^ |
| Education |  |  |
| High School Graduate | Reference | Reference |
| <High School | 2.37(2.16, 2.61) ^**^ | 7.13(6.33, 8.04) ^**^ |
| College | 0.47(0.43, 0.52) ^**^ | 0.26(0.21, 0.32) ^**^ |
| Wealth quartiles |  |  |
| 4^th^ quartile (highest) | Reference | Reference |
| 3^rd^ quartile | 1.18(1.07, 1.32) ^**^ | 1.48(1.19, 1.83) ^**^ |
| 2^nd^ quartile | 1.53(1.38, 1.70) ^**^ | 1.78(1.45, 2.20) ^**^ |
| 1^st^ quartile (lowest) | 1.66(1.48, 1.86) ^**^ | 3.38(2.75, 4.15) ^**^ |
| Smoking |  |  |
| Never smoker | Reference | Reference |
| Past smoker | 0.98(0.91, 1.06) | 0.91(0.81, 1.03) |
| Current smoker | 1.22(1.11, 1.35) ^**^ | 1.12(0.97, 1.29) |
| BMI categories, n (%) |  |  |
| Normal weight | Reference | Reference |
| Underweight | 0.97(0.69, 1.38) | 1.02(0.63, 1.67) |
| Overweight | 0.95(0.87, 1.03) | 0.79(0.69, 0.90) ^**^ |
| Obese | 1.00(0.91, 1.09) | 0.75(0.66, 0.88) ^**^ |

Note: Reference group - low risk with late-life increase. The model was adjusted for baseline age.

^**^p<0.01

1. **Predicted probabilities of cognitive impairment at decades of age in the full group-based trajectory model (GBTM)**

**Table S7. Predicted probabilities of cognitive impairment at decades of age in the full model**

|  |  |  |  |  |  |
| --- | --- | --- | --- | --- | --- |
|  | Age 51 | Age 60 | Age 70 | Age 80 | Age 90 |
| Group 1 |  |  |  |  |  |
| No MM | 0.00(0.00,0.00) | 0.00(0.00,0.01) | 0.01(0.01,0.02) | 0.05(0.04,0.06) | 0.24(0.20,0.27) |
| Somatic MM | 0.00(0.00,0.01) | 0.01(0.00,0.01) | 0.02(0.02,0.02) | 0.07(0.06,0.07) | 0.29(0.26,0.32) |
| Stroke MM | 0.01(0.00,0.01) | 0.01(0.01,0.02) | 0.04(0.03,0.04) | 0.13(0.11,0.15) | 0.45(0.41,0.50) |
| Depressive MM | 0.01(0.00,0.01) | 0.01(0.01,0.02) | 0.04(0.03,0.04) | 0.13(0.11,0.15) | 0.45(0.40,0.50) |
| Stroke & Depressive MM | 0.01(0.00,0.02) | 0.02(0.01,0.03) | 0.07(0.05,0.08) | 0.22(0.17,0.27) | 0.61(0.54,0.68) |
|  |  |  |  |  |  |
| Group 2 |  |  |  |  |  |
| No MM | 0.10(0.08,0.11) | 0.10(0.09,0.11) | 0.17(0.16,0.19) | 0.45(0.42,0.48) | 0.86(0.84,0.89) |
| Somatic MM | 0.14(0.12,0.16) | 0.14(0.13,0.15) | 0.24(0.22,0.26) | 0.55(0.52,0.57) | 0.90(0.89,0.92) |
| Stroke MM | 0.31(0.26,0.35) | 0.31(0.27,0.34) | 0.46(0.42,0.50) | 0.77(0.74,0.79) | 0.96(0.95,0.97) |
| Depressive MM | 0.24(0.20,0.27) | 0.23(0.21,0.26) | 0.37(0.34,0.40) | 0.70(0.67,0.72) | 0.95(0.94,0.96) |
| Stroke & Depressive MM | 0.41(0.35,0.47) | 0.41(0.36,0.46) | 0.57(0.52,0.62) | 0.84(0.81,0.87) | 0.98(0.97,0.98) |
|  |  |  |  |  |  |
| Group 3 |  |  |  |  |  |
| No MM | 0.56(0.52,0.61) | 0.61(0.59,0.64) | 0.78(0.75,0.80) | 0.93(0.91,0.95) | 0.99(0.98,1.00) |
| Somatic MM | 0.64(0.59,0.68) | 0.68(0.66,0.71) | 0.83(0.81,0.85) | 0.95(0.94,0.96) | 0.99(0.99,1.00) |
| Stroke MM | 0.85(0.79,0.90) | 0.87(0.83,0.92) | 0.94(0.91,0.96) | 0.98(0.98,0.99) | 1.00(1.00,1.00) |
| Depressive MM | 0.75(0.71,0.79) | 0.79(0.76,0.81) | 0.89(0.87,0.91) | 0.97(0.96,0.98) | 1.00(0.99,1.00) |
| Stroke & Depressive MM | 0.86(0.79,0.92) | 0.88(0.83,0.93) | 0.94(0.91,0.97) | 0.98(0.98,0.99) | 1.00(1.00,1.00) |

*MM: multimorbidity

**References**

1. Nagin, D.S., *Group-Based Modeling of Development*. 2005, Cambridge, MA: Harvard University Press.

2. Haviland, A.M., Bobby L. Jones, and Daniel S. Nagin., *Group-based trajectory modeling extended to account for nonrandom participant attrition.* Sociological Methods & Research, 2011. **40**(2): p. 367-390.

**6. Technical Appendix**

Visualizing predicted probabilities of cognitive impairment with multimorbidity transition at pre-specified age (60/70/80) requires three steps: (1) Construct the group-based trajectory model with time-varying multimorbidity covariates and conduct post-model estimation for predicted probabilities using a specified set of values for time-varying multimorbidity covariates. (2) Extract the predicted probabilities from post-model estimates to create a dataset used for graphical presentation. (3) Load the dataset to visualize trajectories of predicted probabilities with multimorbidity transition. Details are described below and example codes that can be reproduced are also provided.

1. **Model construction and post-model estimation for predicted probabilities**

In our analyses, we constructed the model and did post-model estimation using the ‘traj’ plugin in STATA version [1]. Time-varying covariates are specified in the ‘traj’ plugin using the ‘tcov’ option. Obtaining predicted probabilities for a specific multimorbidity profile (i.e. a transition occurring at a specified time point) requires that you input a row matrix of values for the multimorbidity covariate profile that you want to visualize into the ‘plottcov’ option of the ‘traj’ command. The ‘plottcov’ option will then calculate the trajectory for each group using the specified set of time-varying covariate values. Details are provided in the documentation for the ‘traj’ Stata plugin [1]. SAS users can also find helpful technical details in the SAS documentation for the ‘ Proc Traj’ software package [2].

In Stata, the ‘plottcov’ option requires the construction of a matrix object to represent a specific covariate profile. With multiple time-varying covariates, the length of the row matrix specified in the ‘plottcov’ option is equal to the number of covariates specified in the ‘tcov’ option and the values must be entered in the same order. In our case, we have a total of 160 variables - 40 variables (age 51-90) for each of four dummy variables (No MM, Stroke MM, Depressive MM, Stroke & Depressive MM, EXCEPT the reference group Somatic MM) specified in the ‘tcov’ option, so we should input a total of 160 values - a set of 40 values of either 1 or 0 for each of four dummy variables in the ‘plottcov’ option to plot the impact of time-varying multimorbidity covariates.

For example, if you want to get the predicted probabilities with transition from Somatic MM to Stroke & Depressive MM at age 70, you need to generate a row matrix of 40 zeros for No MM, 40 zeros for Stroke MM, 40 zeros for Depressive MM, 19 zeros and 21 ones for Stroke & Depressive MM (the Stroke & Depressive MM is “turned on” at 70 – the 20^th^ value for Stroke & Depressive MM changes from 0 to 1). The example STATA code for creating the row matrix of the values described above is shown below:

| clear  *Create a blank dataset with 40 observations  set obs 40  *generate variables for each of the multimorbidity indicators EXCEPT the reference group  *mm_no - No MM, mm_ss – Stroke MM, mm_sd – Depressive MM, mm_ssd – Stroke and Depressive *MM and set them all to 0  gen mm_no=0  gen mm_ss=0  gen mm_sd=0  gen mm_ssd=0  *Since age is centered at age 51, we 'turn on' ssd multimorbidity by setting everything from 20+ to 1  replace mm_ssd=1 if _n>=20  *Output each variable in the dataset as a column vector (the default)  mkmat mm_no mm_ss mm_sd mm_ssd  *Loop over each vector to transpose them.  foreach m in "mm_no" "mm_ss" "mm_sd" "mm_ssd"{  mat `m'=`m''  }  *Merge the resulting row vectors  mat ssd_70=(mm_no,mm_ss,mm_sd,mm_ssd)  *Print the row matrix ‘ssd_70’ that we need to put into plottov to represent transition from Somatic MM to Stroke & Depressive MM at age 70  matrix list ssd_70 |
| --- |

This same approach can be used to generate a row matrix of values for different covariate profiles -- e.g. you could also create a row matrix to show transition from Somatic MM to Stroke MM at age 60/70/80 (or any specified age) or transition from Somatic MM to Depressive MM at 60/70/80 (or any specified age).

1. **Extract the predicted probabilities from post-model estimates to create a dataset used for graphical presentation.**

In the first step, we created ‘ssd_70’ - a row matrix of values to represent transition from Somatic MM to Stroke & Depressive MM at age 70. Now we want to input the row matrix of values in plottcov command to obtain the post-model estimates of predicted probabilies of cognitive impairment. However, the post-model estimates are stored in a matrix that is used to create the plots in Stata. Thus, we need to extract this matrix of predicted probabilities and store them into a dataset for graphical presentation using R. The example STATA code is shown below:

| *Fit trajectory model. Note that the matrix specifying somatic only is named in the plottcov option.  traj, var(cind_dem_51-cind_dem_90) indep (age_c_51-age_c_90) model (logit) order (2 2 2) tcov (mm_no_51-mm_no_90 mm_ss_51-mm_ss_90 mm_sd_51-mm_sd_90 mm_ssd_51-mm_ssd_90)  plottcov(ssd_70)  *Save the results to a dataset to plot in R. First step is to extract the values used to create the plot. They are stored in the matrix e(plot1).  *Write stored matrix to matrix ssd  mat ssd=e(plot1)  *Clear environment  clear  *Create a blanck dataset with 40 observations  set obs 40  *Use the svmat command to create a dataset from a matrix. Specify that the variablenames are the column names  svmat ssd, names(col)  *Keep the columns that correspond to the estimated trajectory value at each age  keep Est1-Est3  *Generate a variable for age and un-center it  gen age=_n+50  *Make all lowercase  renvars, lower  *Reshape to long form so that it can be used in ggplot2.  *Variables indicating traj-group and age  *est – predicted probabilities  reshape long est, i(age) j(traj_group)  save “plot_data_ssd_70.dta”, replace |
| --- |

1. **Visualize trajectories of predicted probabilities with multimorbidity transition using the dataset that was created.**

Data visualizations of predicted trajectories were constructed using the ‘ggplot2’ package[3] in R 3.6.2. Other graphical packages and software can also provide visualization using the dataset. Using “plot_data_ssd_70.dta” that was created in the example codes above, you can visualize trajectories of predicted probabilities with transition from Somatic MM to Stroke & Somatic MM for each trajectory group. You can also append different created datasets to compare between trajectories with different covariate profiles, e.g. you could compare 1) Somatic MM from 51 to 90, 2) Transition from Somatic MM to Stroke MM at age 70, 3) Transition from Somatic MM to Depressive MM at age 70, and 4) Transition from Somatic MM to Stroke & Depressive MM at age 70 all in the same graph.

**References**

1. L. Jones, Bobby; Nagin, Daniel (2018): A Stata Plugin for Estimating Group-Based Trajectory Models. Carnegie Mellon University. Journal contribution. <https://doi.org/10.1184/R1/6470963.v1>
2. Jones, B.L. SAS documentation for PROC TRAJ. 2022 [cited 2023; Available from: <https://www.andrew.cmu.edu/user/bjones/documentation.htm>.
3. Wickham H (2016). ggplot2: Elegant Graphics for Data Analysis. Springer-Verlag New York. ISBN 978-3-319-24277-4, https://ggplot2.tidyverse.org.
