# Supplementary material for: Mental-somatic multimorbidity in trajectories of cognitive function for middle-aged and older adults
Source: PLoS One. 2024 May 14;19(5):e0303599. doi: 10.1371/journal.pone.0303599 (PMC11093294; doi:10.1371/journal.pone.0303599)
Supplement: S2 Appendix — (DOCX) [file pone.0303599.s002.docx]

**S2 Appendix.** Technical details and reproducible codes for visualization of cognitive impairment trajectories with multimorbidity transition.

Visualizing predicted probabilities of cognitive impairment with multimorbidity transition at pre-specified age (60/70/80) requires three steps: (1) Construct the group-based trajectory model with time-varying multimorbidity covariates and conduct post-model estimation for predicted probabilities using a specified set of values for time-varying multimorbidity covariates. (2) Extract the predicted probabilities from post-model estimates to create a dataset used for graphical presentation. (3) Load the dataset to visualize trajectories of predicted probabilities with multimorbidity transition. Details are described below and example codes that can be reproduced are also provided.

1. **Model construction and post-model estimation for predicted probabilities**

In our analyses, we constructed the model and did post-model estimation using the ‘traj’ plugin in STATA version [1]. Time-varying covariates are specified in the ‘traj’ plugin using the ‘tcov’ option. Obtaining predicted probabilities for a specific multimorbidity profile (i.e. a transition occurring at a specified time point) requires that you input a row matrix of values for the multimorbidity covariate profile that you want to visualize into the ‘plottcov’ option of the ‘traj’ command. The ‘plottcov’ option will then calculate the trajectory for each group using the specified set of time-varying covariate values. Details are provided in the documentation for the ‘traj’ Stata plugin [1]. SAS users can also find helpful technical details in the SAS documentation for the ‘ Proc Traj’ software package [2].

In Stata, the ‘plottcov’ option requires the construction of a matrix object to represent a specific covariate profile. With multiple time-varying covariates, the length of the row matrix specified in the ‘plottcov’ option is equal to the number of covariates specified in the ‘tcov’ option and the values must be entered in the same order. In our case, we have a total of 160 variables - 40 variables (age 51-90) for each of four dummy variables (No MM, Stroke MM, Depressive MM, Stroke & Depressive MM, EXCEPT the reference group Somatic MM) specified in the ‘tcov’ option, so we should input a total of 160 values - a set of 40 values of either 1 or 0 for each of four dummy variables in the ‘plottcov’ option to plot the impact of time-varying multimorbidity covariates.

For example, if you want to get the predicted probabilities with transition from Somatic MM to Stroke & Depressive MM at age 70, you need to generate a row matrix of 40 zeros for No MM, 40 zeros for Stroke MM, 40 zeros for Depressive MM, 19 zeros and 21 ones for Stroke & Depressive MM (the Stroke & Depressive MM is “turned on” at 70 – the 20^th^ value for Stroke & Depressive MM changes from 0 to 1). The example STATA code for creating the row matrix of the values described above is shown below:

| clear  *Create a blank dataset with 40 observations  set obs 40  *generate variables for each of the multimorbidity indicators EXCEPT the reference group  *mm_no - No MM, mm_ss – Stroke MM, mm_sd – Depressive MM, mm_ssd – Stroke and Depressive *MM and set them all to 0  gen mm_no=0  gen mm_ss=0  gen mm_sd=0  gen mm_ssd=0  *Since age is centered at age 51, we 'turn on' ssd multimorbidity by setting everything from 20+ to 1  replace mm_ssd=1 if _n>=20  *Output each variable in the dataset as a column vector (the default)  mkmat mm_no mm_ss mm_sd mm_ssd  *Loop over each vector to transpose them.  foreach m in "mm_no" "mm_ss" "mm_sd" "mm_ssd"{  mat `m'=`m''  }  *Merge the resulting row vectors  mat ssd_70=(mm_no,mm_ss,mm_sd,mm_ssd)  *Print the row matrix ‘ssd_70’ that we need to put into plottov to represent transition from Somatic MM to Stroke & Depressive MM at age 70  matrix list ssd_70 |
| --- |

This same approach can be used to generate a row matrix of values for different covariate profiles -- e.g. you could also create a row matrix to show transition from Somatic MM to Stroke MM at age 60/70/80 (or any specified age) or transition from Somatic MM to Depressive MM at 60/70/80 (or any specified age).

1. **Extract the predicted probabilities from post-model estimates to create a dataset used for graphical presentation.**

In the first step, we created ‘ssd_70’ - a row matrix of values to represent transition from Somatic MM to Stroke & Depressive MM at age 70. Now we want to input the row matrix of values in plottcov command to obtain the post-model estimates of predicted probabilies of cognitive impairment. However, the post-model estimates are stored in a matrix that is used to create the plots in Stata. Thus, we need to extract this matrix of predicted probabilities and store them into a dataset for graphical presentation using R. The example STATA code is shown below:

| *Fit trajectory model. Note that the matrix specifying somatic only is named in the plottcov option.  traj, var(cind_dem_51-cind_dem_90) indep (age_c_51-age_c_90) model (logit) order (2 2 2) tcov (mm_no_51-mm_no_90 mm_ss_51-mm_ss_90 mm_sd_51-mm_sd_90 mm_ssd_51-mm_ssd_90)  plottcov(ssd_70)  *Save the results to a dataset to plot in R. First step is to extract the values used to create the plot. They are stored in the matrix e(plot1).  *Write stored matrix to matrix ssd  mat ssd=e(plot1)  *Clear environment  clear  *Create a blanck dataset with 40 observations  set obs 40  *Use the svmat command to create a dataset from a matrix. Specify that the variablenames are the column names  svmat ssd, names(col)  *Keep the columns that correspond to the estimated trajectory value at each age  keep Est1-Est3  *Generate a variable for age and un-center it  gen age=_n+50  *Make all lowercase  renvars, lower  *Reshape to long form so that it can be used in ggplot2.  *Variables indicating traj-group and age  *est – predicted probabilities  reshape long est, i(age) j(traj_group)  save “plot_data_ssd_70.dta”, replace |
| --- |

1. **Visualize trajectories of predicted probabilities with multimorbidity transition using the dataset that was created.**

Data visualizations of predicted trajectories were constructed using the ‘ggplot2’ package[3] in R 3.6.2. Other graphical packages and software can also provide visualization using the dataset. Using “plot_data_ssd_70.dta” that was created in the example codes above, you can visualize trajectories of predicted probabilities with transition from Somatic MM to Stroke & Somatic MM for each trajectory group. You can also append different created datasets to compare between trajectories with different covariate profiles, e.g. you could compare 1) Somatic MM from 51 to 90, 2) Transition from Somatic MM to Stroke MM at age 70, 3) Transition from Somatic MM to Depressive MM at age 70, and 4) Transition from Somatic MM to Stroke & Depressive MM at age 70 all in the same graph.

**References**

1. L. Jones, Bobby; Nagin, Daniel (2018): A Stata Plugin for Estimating Group-Based Trajectory Models. Carnegie Mellon University. Journal contribution. <https://doi.org/10.1184/R1/6470963.v1>
2. Jones, B.L. SAS documentation for PROC TRAJ. 2022 [cited 2023; Available from: <https://www.andrew.cmu.edu/user/bjones/documentation.htm>.
3. Wickham H (2016). ggplot2: Elegant Graphics for Data Analysis. Springer-Verlag New York. ISBN 978-3-319-24277-4, https://ggplot2.tidyverse.org.
